# Long noncoding RNA MCM3AP‐AS1 enhances cell proliferation and metastasis in colorectal cancer by regulating miR‐193a‐5p/SENP1

**DOI:** 10.1002/cam4.3830

**Published:** 2021-03-08

**Authors:** Mingyue Zhou, Zehua Bian, Bingxin Liu, Yi Zhang, Yulin Cao, Kaisa Cui, Shengbai Sun, Jiuming Li, Jia Zhang, Xue Wang, Chaoqun Li, Surui Yao, Yuan Yin, Bojian Fei, Zhaohui Huang

**Affiliations:** ^1^ Department of Gastrointestinal Surgery Affiliated Hospital of Jiangnan University Wuxi, Jiangsu China; ^2^ Wuxi Cancer Institute Affiliated Hospital of Jiangnan University Wuxi, Jiangsu China; ^3^ Cancer Epigenetics Program Wuxi School of Medicine Jiangnan University Wuxi, Jiangsu China; ^4^ Department of Colorectal Surgery The First Affiliated Hospital of Nanjing Medical University Nanjing Jiangsu China

**Keywords:** colorectal cancer, long noncoding RNAs, MCM3AP antisense RNA 1, microRNAs, SENP1

## Abstract

**Background:**

Accumulating evidences have shown that long noncoding RNAs (lncRNAs) play key roles in many diseases, including cancer. Several studies reported that MCM3AP antisense RNA 1 (MCM3AP‐AS1) was associated with the tumorigenesis and progression. However, the specific function and mechanism of MCM3AP‐AS1 in colorectal cancer (CRC) have not been fully understood.

**Methods:**

The expression of MCM3AP‐AS1 was detected by quantitative reverse transcription PCR (RT‐qPCR) in CRC tissues and matched noncancerous tissues (NCTs). CCK‐8 assay, colony formation assay, transwell assay, xenograft and lung metastasis mouse models were used to examine the tumor‐promoting function of MCM3AP‐AS1 *in vitro* and *in vivo*. The binding relationship between MCM3AP‐AS1, miR‐193a‐5p and sentrin‐specific peptidase 1 (SENP1) were screened and identified by databases, RT‐qPCR, dual luciferase reporter assay and western blot.

**Results:**

In the present study, we got that the expression of MCM3AP‐AS1 was higher in CRC tissues than in paired NCTs, and increased MCM3AP‐AS1 expression was associated with adverse outcomes in CRC patients. Functional experiments *in vitro* revealed that silencing of MCM3AP‐AS1 could inhibit the proliferation, colony formation, migratory, and invasive abilities of CRC cells. The mouse models of xenograft and lung metastasis further confirmed that *in vivo* silencing MCM3AP‐AS1 could significantly inhibit the growth and metastasis of CRC. Further mechanism studies indicated that MCM3AP‐AS1 could sponge miR‐193a‐5p and inhibit the activity of it. What is more, SENP1 was proved to be a novel target of miR‐193a‐5p and could be upregulated by MCM3AP‐AS1. At last, we observed that SENP1 overexpression in CRC tissues was closely related to unfavorable prognosis.

**Conclusion:**

Taken together, we identified in CRC the MCM3AP‐AS1/miR‐193a‐5p/SENP1 regulatory axis, which affords a therapeutic possibility for CRC.

## Graphical abstract

MCM3AP‐AS1 functions as an oncogene in several cancers. We reported that MCM3AP‐AS1 is up‐regulated in colorectal cancer tissues and indicates poor prognosis. We revealed its tumor‐promoting role *in vitro* and *in vivo* by promoting colorectal cancer cell proliferation and metastasis. Further mechanistic investigations indicated that MCM3AP‐AS1 exerts oncogene function with a novel mechanism by sponging miR‐193a‐5p and up‐regulating its target gene SENP1. These data suggest that MCM3AP‐AS1 can serve as potential therapeutic target for colorectal cancer treatment.

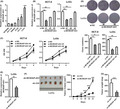

## 1 INTRODUCTION

Colorectal cancer (CRC) is the third most common cancer and the second leading cause of the cancer‐related death in the world. In China, the incidence rate and mortality rate of CRC are also increasing every year.^1^ The development of CRC is a multistep process, involving a series of complex genetic and epigenetic changes, but its mechanism has not been fully elucidated.^2^, ^3^ Therefore, it is extremely urgent to clarify the pathogenesis and search for new treatment method.

Long noncoding RNAs (lncRNAs) are functionally defined as a new type of noncoding RNA (ncRNA). They are more than 200 nucleotides in length and have no protein coding potential.^4^, ^5^ LncRNAs have become a new research hotspot due to their large number, rich functions, and diverse mechanisms.^6^ LncRNAs are involved in epigenetic regulation, DNA damage, microRNAs (miRNAs) sponges, and some other biological processes of tumors.^7^ We have reported some lncRNAs that regulate CRC growth, metastasis, and chemoresistance and may be potential prognostic biomarkers or therapeutic targets.^8^, ^9^, ^10^, ^11^, ^12^, ^13^, ^14^, ^15^ For example, we previously reported that FEZF1‐AS1 promotes CRC growth and metastasis by increasing PKM2 activity.^8^ We also found that LINC00152 promotes CRC proliferation, metastasis, and chemoresistance by binding with miR‐139‐5p,^10^ whereas UCA1 promotes CRC proliferation and chemotherapy resistance by inhibiting miR‐204‐5p.^11^ Recently, Liu et al. reported that knockdown of HOTAIR could enhance radiosensitivity by regulating miR‐93 and ATG12 in CRC.^16^ All these studies indicate that lncRNAs have key roles in the development of CRC.

MCM3AP antisense RNA 1 (MCM3AP‐AS1) has been reported for the first time to promote angiogenesis in glioblastoma.^17^ It was then confirmed to play a carcinogenic role in gastric cancer, breast cancer, pancreatic cancer, and hepatocellular carcinoma.^18^, ^19^, ^20^, ^21^ All these studies reveal the significant roles of MCM3AP‐AS1 in human cancers. However, its role in CRC was still not fully investigated, and the mechanism remains to be elucidated.

## 2 MATERIALS AND METHODS

### 2.1 Clinical sample

A total of 131 human primary CRC tissues and their paired adjacent noncancerous tissues (NCTs) were obtained from Affiliated Hospital of Jiangnan University (Table S1). All patients have signed informed consent and the project was approved by the Clinical Research Ethics Committees of the Affiliated Hospital of Jiangnan University.

### 2.2 Cell lines and culture

Human CRC cell lines HCT‐8, HCT116, LoVo, HT29, and SW620 were purchased from the American Type Culture Collection (ATCC). LoVo was cultured in F12 K medium, and the other cells were maintained with DMEM medium which contains 10% fetal bovine serum. All these cell lines were cultured in 5% CO_2_ at 37°C.

### 2.3 Plasmids and transfection

The siRNA of MCM3AP‐AS1, miR‐193a‐5p mimics and miR‐193a‐5p inhibitor were ordered from Shanghai Genepharma Co., Ltd. Full length of sentrin‐specific peptidase 1 (SENP1) was cloned into pcDNA3.1 (+). The short‐hairpin MCM3AP‐AS1‐#1 was constructed into pLKO.1‐shRNA vector for lentivirus package. The transfection of these plasmids was completed by using lipofectamine 2000 (Invitrogen, CA, USA) according to the manufacturer's instructions.

### 2.4 Quantitative reverse transcription PCR

Total RNA of cells or tissue specimens was extracted using RNA isolater (Vazyme, Jiangsu, China) and reverse transcribed into cDNA using HiFiScript cDNA Synthesis Kit (CWBIO, Beijing, China). MCM3AP‐AS1 and SENP1 expression levels were measured by RT‐qPCR using UltraSYBR Mixture (CWBIO) on an ABI system (Thermo Fisher Scientific, MA, USA) with the program of 95°C for 10 min, 40 cycles of 95°C for 15 s and 60°C for 1 min. The expression levels of MCM3AP‐AS1 were determined using RT‐qPCR with β‐actin as an internal control. The relative gene expression levels were calculated by 2^−△△Ct^. The related sequences are listed in Table S2.

### 2.5 Cell Counting Kit 8 and colony formation assays

The capacity of cell proliferation was measured by CCK‐8 (Beyotime, Shanghai, China) and colony formation assay. These assays were carried out as we described previously.^8^

### 2.6 Transwell assay

About 1 × 10^5^ LoVo cells or 6 × 10^4^ HCT‐8 cells were added into the upper compartment of transwell chamber (Corning, NY, USA). After 48‐h incubation, cells on the lower surface were fixed with 10% formaldehyde for half an hour and then stained with crystal violet for the following observation. In invasion assay, matrigel (BD Biosciences, CA, USA) was used to coat the transwell chamber before the experiments.

### 2.7 Xenograft mouse model

A total of 1.5 × 10^6^ LoVo cells stably transfected with sh‐MCM3AP‐AS1 or sh‐Ctrl lentiviral vector were subcutaneously injected to the different flank of 4‐week‐old male BALB/c nude mice (Shanghai SLAC Laboratory Animal Co. Ltd, Shanghai, China) (*n* = 5 for each group). The tumor formation was observed every 3 days, and the tumor size was measured. For the in vivo metastasis model, 2 × 10^6^ cells were injected into the 7‐week‐old male BALB/c nude mice (*n* = 5 for each group) via the tail vein. Five weeks after injection, the lung nodules of mice were observed to measure the capability of tumor metastasis. All the animal experiments were approved by the Clinical Research Ethics Committees of the Affiliated Hospital of Jiangnan University.

### 2.8 Dual luciferase reporter gene assay

The luciferase reporter vectors of MCM3AP‐AS1 and SENP1 which contain wild‐type (WT) or mutated (MUT) miRNA binding sites were constructed. Luciferase reporter plasmids were cotransfected with miR‐193a‐5p mimics and miR‐NC mimics into 293 T and HCT‐8 cells by Lipofectamine 2000. The luciferase activities of these cells were detected at 48 h after transfection using the Dual‐Luciferase® Reporter Assay System (Beyotime).

### 2.9 Western blot

After 48 h of transfection, the cells were lysed in RIPA buffer (Beyotime) supplemented with protease inhibitors cocktail (MCE, New Jersey, USA). Then the protein was separated by 10% SDS‐PAGE and transferred to a PVDF membrane (Millipore, Burlington, USA). After being blocked in 5% skimmed milk powder for 2 h, the membranes were incubated with primary antibodies against SENP1 (1:1000, Proteintech, Chicago, USA), β‐actin (1:5000, ABclonal, Boston, USA) or GAPDH (1:5000, ABclonal) overnight at 4℃. The protein band intensity was detected using ChemiDOCTMXRS^+^ imaging system (BIO‐RAD, Minnesota, USA).

#### 2.9.1 Immunohistochemistry

The slides of tissue microarray were incubated with the primary antibody SENP1 (1:100, Proteintech) overnight at 4℃. IHC was performed as we described previously.^8^

#### 2.9.2 RNA immunoprecipitation

EZ‐Magna RIP kit (Millipore) was used for a RIP assay as we previously described.^10^

#### 2.9.3 Statistical analysis

The data were analyzed by GraphPad Prism version 8.0 (GraphPad Software, California, USA), SPSS 20 software (SPSS, NY, USA). All results are presented as the mean values ± SD. Student's *t* test and *χ* ^2^ test was used to compare the significant differences between groups. The differences in survival rates were determined by the Kaplan‐Meier method and compared by the log‐rank test. *p* < 0.05 was considered to indicate statistically significant.

## 3 RESULTS

### 3.1 MCM3AP‐AS1 is up‐regulated in CRC tissues and indicates poor prognosis

**FIGURE 1:**
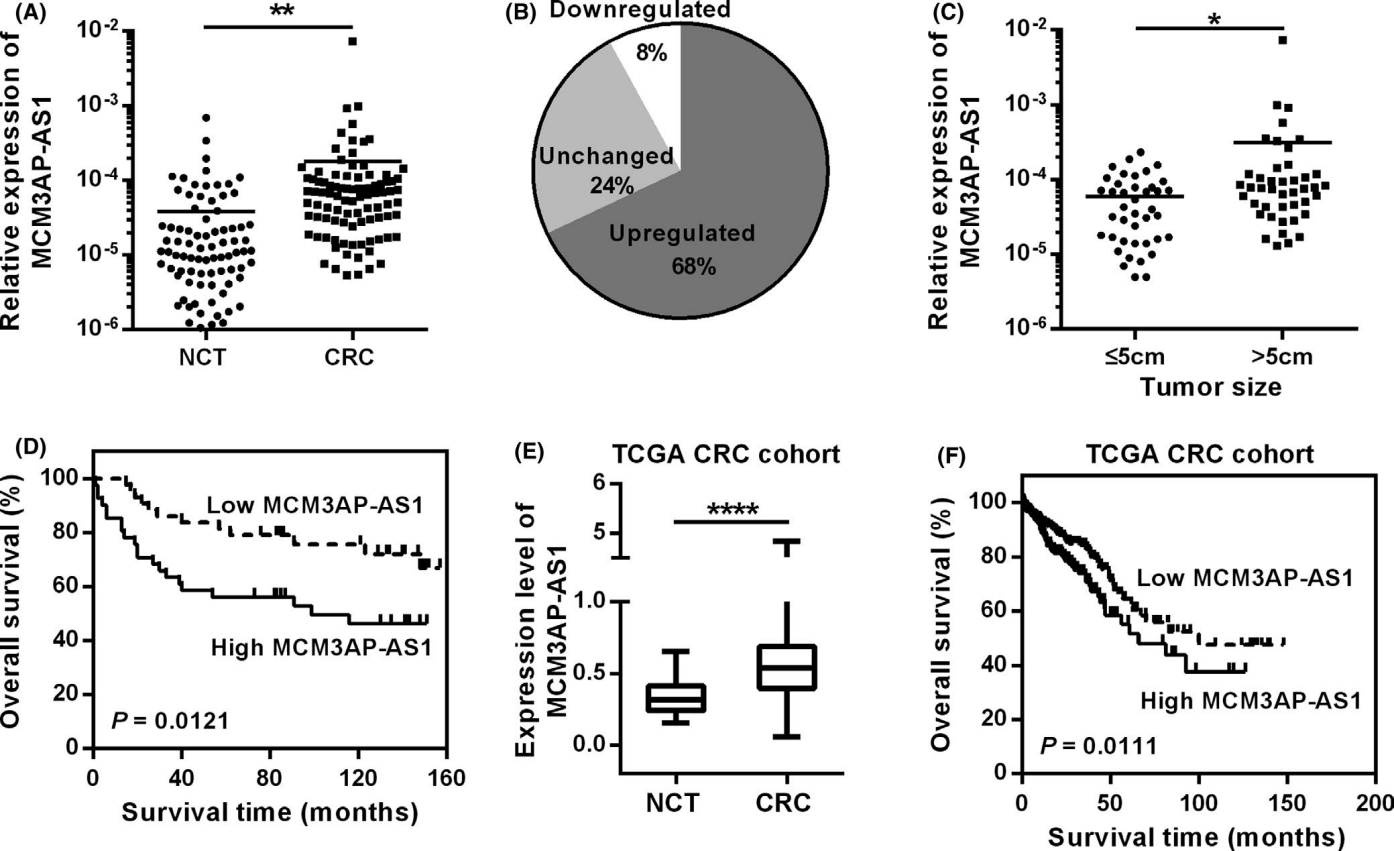
Up regulation of MCM3AP‐AS1 indicates poor clinical outcome in CRC. (A,B) RT‐qPCR was used to detect the relative expression of MCM3AP‐AS1 in CRC tissues and NCTs. (C) The expression of MCM3AP‐AS1 was related to tumor size. (D) Kaplan–Meier survival analysis of CRC patients with different expression level of MCM3AP‐AS1. (E) The expression level of MCM3AP‐AS1 in TCGA CRC dataset. (F) The Kaplan‐Meier curve of MCM3AP‐AS1 of CRC in TCGA database. *p < 0.05, **p < 0.01, ****p < 0.0001.

We used RT‐qPCR to detect the expression level of MCM3AP‐AS1 in CRC tissues. As shown in Figure 1A,B, MCM3AP‐AS1 was remarkably up‐regulated in CRC tissues (Figure 1A,B). Moreover, elevated expression level of MCM3AP‐AS1 was associated with larger tumor size and indicated unfavorable survival (Figure 1C,D). The TCGA database and GEO GSE21510 database also showed that MCM3AP‐AS1 is significantly up‐regulated in CRC and associated with worse outcome of CRC patients (Figure 1E,F and Figure S1). Taken together, our results showed that MCM3AP‐AS1 is up‐regulated in CRC, which is associated with poor clinical outcome.

### 3.2 MCM3AP‐AS1 promotes CRC cell proliferation and metastasis

**FIGURE 2:**
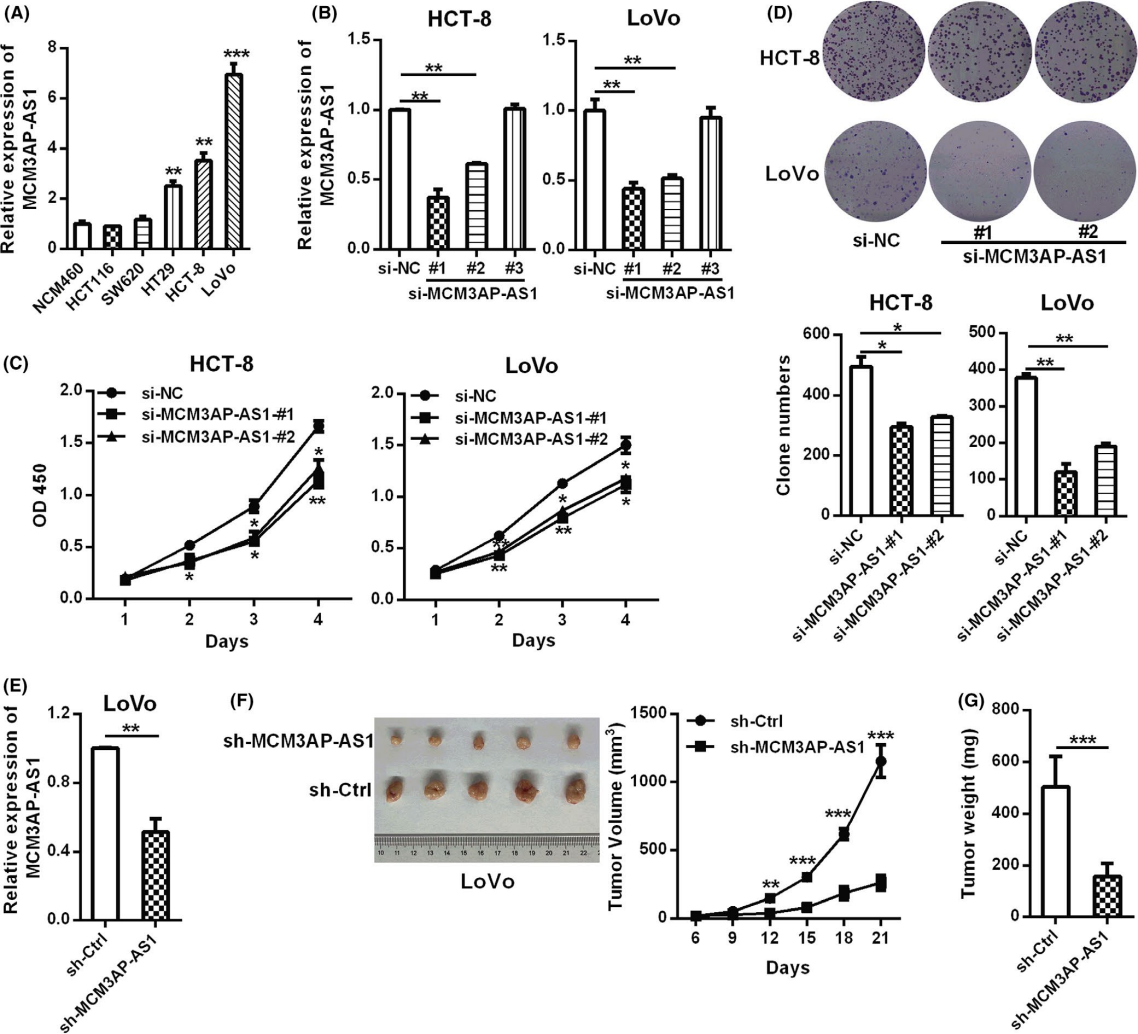
MCM3AP‐AS1 promoted CRC proliferation. (A) RT‐qPCR was applied to detect the relative expression levels of MCM3AP‐AS1 in CRC cell lines. (B) RT‐qPCR was used to detect the knockdown efficiency of three different MCM3AP‐AS1 siRNAs. (C, D) The CCK8 method and colony formation test were used to evaluate the effects of MCM3AP‐AS1 knockdown on cell proliferation. (E) The stable knockdown efficiency of MCM3AP‐AS1 in LoVo cells. (F, G) LoVo cells with MCM3AP‐AS1 stable silencing were injected subcutaneously into nude mice (*n* = 5) to detect the effect of MCM3AP‐AS1 on tumor formation. The tumor volumes (F) were measured every 3 days because the tumor can be obviously observed and the tumor weight (G) were recorded after sacrifice. *p < 0.05, **p < 0.01, ***p < 0.001.

In order to study the role of MCM3AP‐AS1 in CRC cells, we first detected the relative expression of MCM3AP‐AS1 in HCT‐8, HCT116, LoVo, HT29, and SW620 cells by RT‐qPCR. MCM3AP‐AS1 expression level was relative higher in HCT‐8 and LoVo cell lines (Figure 2A). Therefore, we used three independent siRNAs to knock down the expression of MCM3AP‐AS1 and found that siRNA‐#1 and ‐#2 could effectively inhibit the expression of MCM3AP‐AS1 (Figure 2B). CCK8 assays showed that down‐regulation of MCM3AP‐AS1 significantly attenuated cell proliferation of HCT‐8 and LoVo cells (Figure 2C). The same effects were also demonstrated by colony formation assays (Figure 2D). To further validate these effects*in vivo*, we injected MCM3AP‐AS1‐silencing LoVo cells subcutaneously into nude mice. Compared with the control group, tumor growth and tumor weight were dramatically reduced in MCM3AP‐AS1 knockdown group (Figure 2E–G).

**FIGURE 3:**
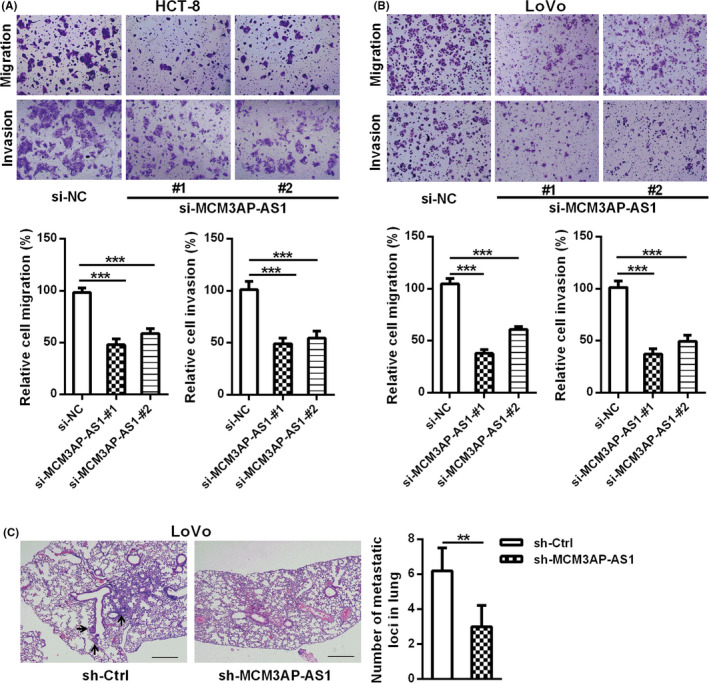
MCM3AP‐AS1 knockdown suppressed CRC cell migration and invasion*in vitro* and metastasis *in vivo*. (A, B) The effects of MCM3AP‐AS1 knockdown on the migration and invasion of HCT‐8 (A) and LoVo (B) cells were detected by transwell assays. (C) MCM3AP‐AS1 knockdown inhibited CRC metastasis. LoVo cells with MCM3AP‐AS1 knockdown were injected to the tail veins of nude mice (*n* = 5) to evaluated the function of MCM3AP‐AS1 in a lung metastasis mouse model. Representative HE‐stained sections of lung tissues are showed in this panel. The number of lung nodules were quantified after dissection. **p < 0.01, ***p < 0.001.

Moreover, the down‐regulation of MCM3AP‐AS1 expression remarkably inhibited cell migration and invasion ability in HCT‐8 and LoVo cells (Figure 3A,B). We also evaluated the functional role of MCM3AP‐AS1 in CRC metastasis using a mouse lung metastasis model. As shown in Figure 3C, the number of lung metastatic tumor nodules in sh‐MCM3AP‐AS1 group was lower than that in control group. All these above results suggested that MCM3AP‐AS1 could promote CRC proliferation and metastasis.

### 3.3 MCM3AP‐AS1 sponges miR‐193a‐5p in CRC

**FIGURE 4:**
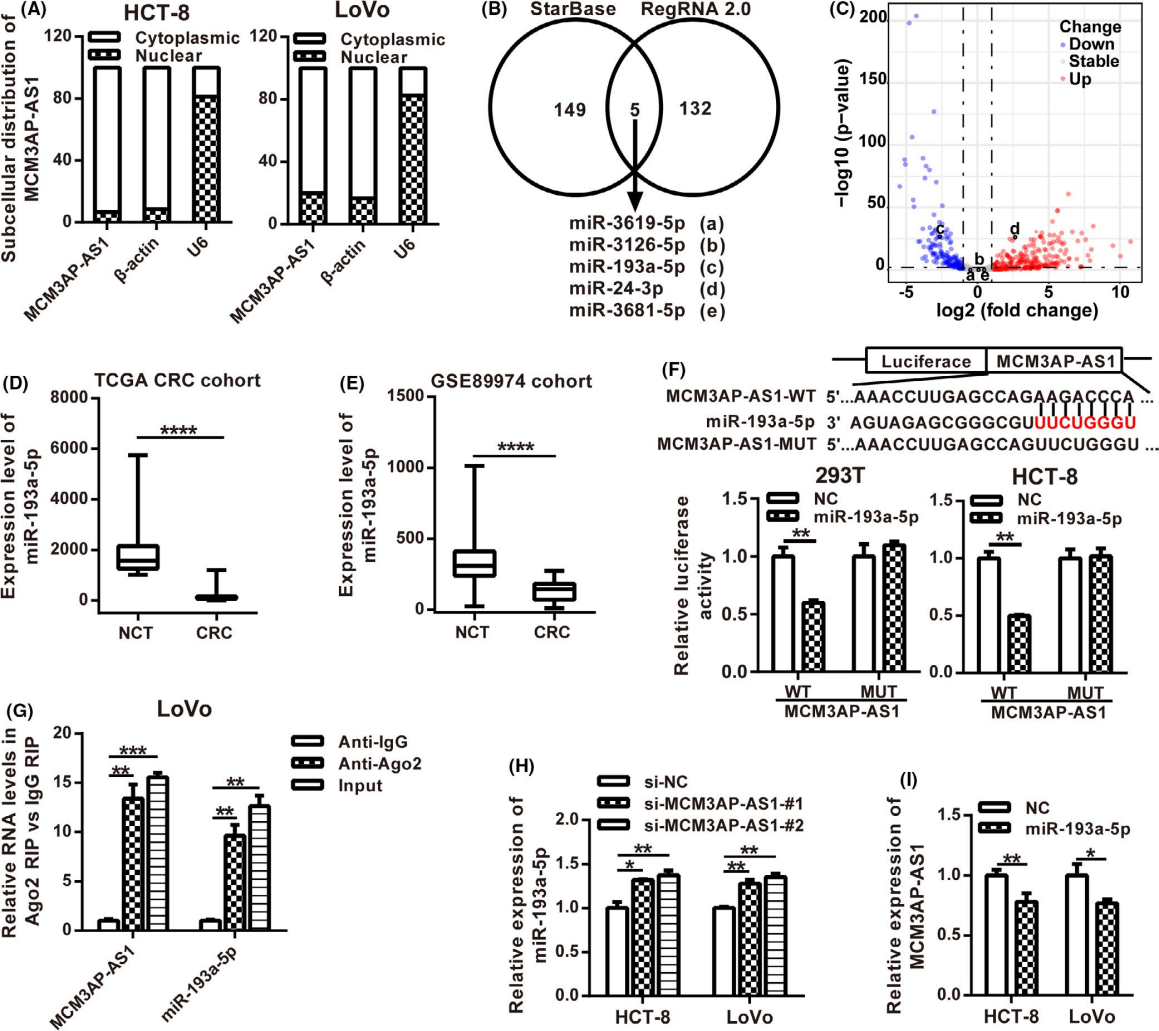
MCM3AP‐AS1 could directly bind to miR‐193a‐5p. (A) Subcellular localization of MCM3AP‐AS1 was detected in HCT‐8 and LoVo cell lines. (B) StarBase and RegRNA 2.0 were used to predict the MCM3AP‐AS1‐associated miRNAs. (C) Volcano maps of differentially expressed miRNAs from TCGA. Differentially expressed miRNAs were distinguished according to |log_2_ (fold change)| >1.0 and *p* value <0.05. Red plots represent up‐regulated miRNAs, blue plots stand for down‐regulated miRNAs, and gray plots represent miRNAs with no significant difference. D,E, The RNA expression levels of miR‐193a‐5p of CRC in TCGA database (D) and GEO GSE89974 database (E). (F) The sequence of MCM3AP‐AS1 was cloned into pLuc vector. The relative luciferase activity of MCM3AP‐AS1‐WT or MCM3AP‐AS1‐MUT cotransfected with miR‐193a‐5p mimic was determined by dual luciferase reporter assay. (G) Cellular lysates from LoVo cells were used for RIP with an Ago2 antibody. The levels of MCM3AP‐AS1 and miR‐193a‐5p were detected by RT‐qPCR. (H) The expression level of miR‐193a‐5p was determined in MCM3AP‐AS1‐depleted HCT‐8 and LoVo cells by RT‐qPCR. (I) The expression level of MCM3AP‐AS1 was examined in miR‐193a‐5p overexpression HCT‐8 and LoVo cells by RT‐qPCR. **p < 0.01, ****p < 0.0001.

In order to explore the mechanism of MCM3AP‐AS1 in CRC, we first detected the subcellular localization of it. The results of RT‐qPCR demonstrated that the cytoplasm is the main localization of MCM3AP‐AS1 in HCT‐8 and LoVo cells, indicating that MCM3AP‐AS1 may play a regulatory role at the post‐transcriptional level through the competitive endogenous RNA mechanism (Figure 4A). In order to search the potential miRNAs that may bind to MCM3AP‐AS1, we compared the miRNAs predicted by starBase and RegRNA 2.0 (Figure 4B). Of the five miRNAs selected by both the two tools, miR‐193a‐5p, which is down‐regulated markedly in CRC, was selected for further study (Figure 4C–E). To prove whether MCM3AP‐AS1 could bind to miR‐193a‐5p, a luciferase reporter vector containing miR‐193a‐5p binding sites was constructed. The results of double luciferase reporter assay showed that miR‐193a‐5p could directly bind to MCM3AP‐AS1‐WT but could not bind to MCM3AP‐AS1‐MUT (Figure 4F). RIP experiments were also performed to verify that whether MCM3AP‐AS1 and miR‐193a‐5p are associated through miRNA ribonucleoprotein complexes (miRNPs). The results of RT‐qPCR showed that MCM3AP‐AS1 and miR‐193a‐5p were enriched in the Ago2‐containing miRNPs (Figure 4G). We also found that the expression of miR‐193a‐5p increased in MCM3AP‐AS1‐depleted CRC cells, whereas MCM3AP‐AS1 was down‐regulated in CRC cells transfected with miR‐193a‐5p mimic (Figure 4H,I). All these results suggest that MCM3AP‐AS1 sponges and regulates miR‐193a‐5p in CRC.

### 3.4 SENP1 is the target of MCM3AP‐AS1/miR‐193a‐5p axis

**FIGURE 5:**
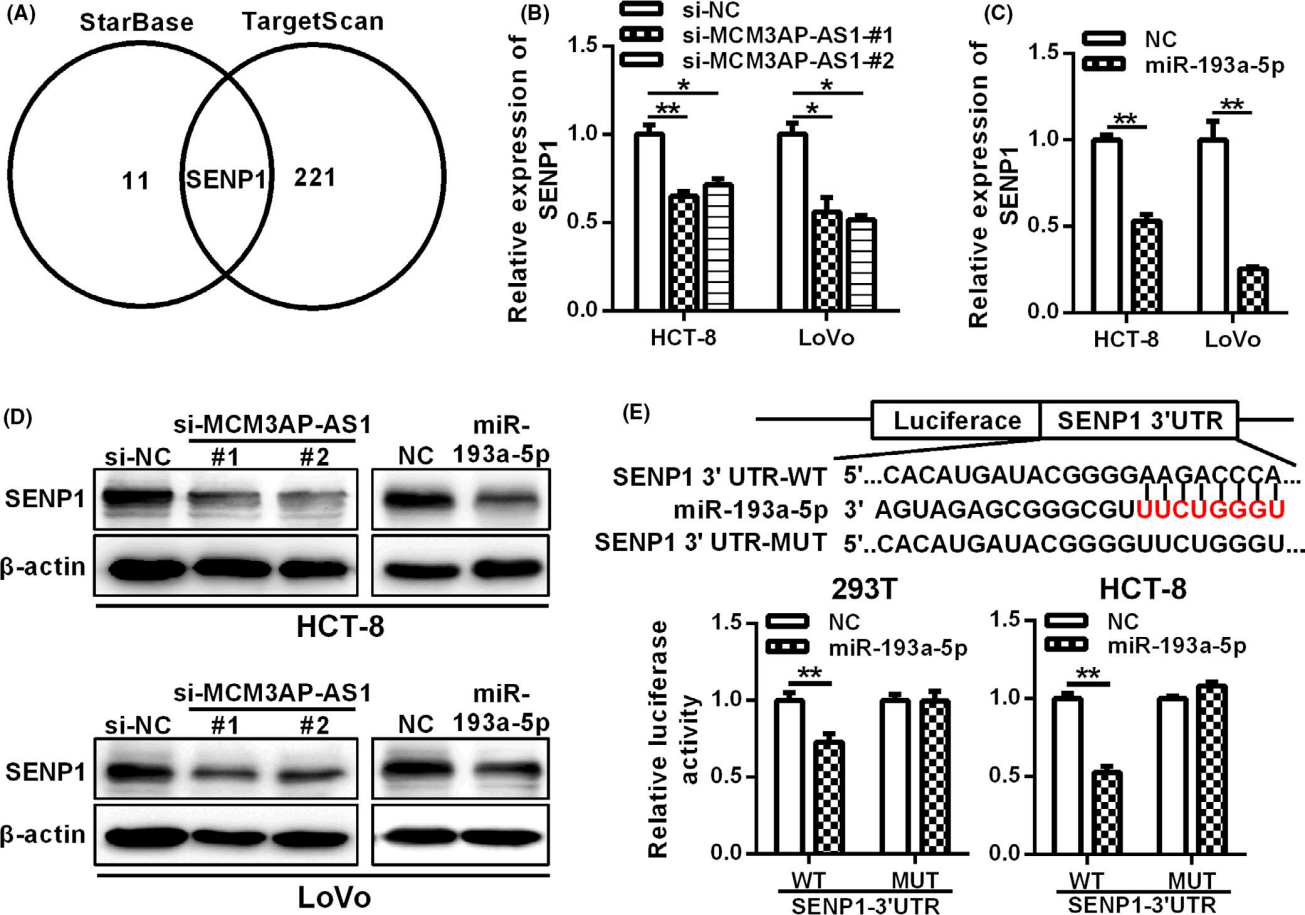
SENP1 is a target of MCM3AP‐AS1/miR‐193a‐5p axis. (A) The potential mRNA targets of MCM3AP‐AS1 and miR‐193a‐5p were predicted by starBase database and targetScan database, respectively. B‐D, The mRNA and protein levels of SENP1 were determined in MCM3AP‐AS1‐depleted or miR‐193a‐5p overexpression CRC cells by RT‐qPCR (B, C) and western blot (D), respectively. (E) The relative luciferase activity of SENP1‐3’UTR‐WT or SENP1‐3’UTR‐MUT cotransfected with miR‐193a‐5p mimics were determined by dual luciferase reporter assays. *p < 0.05, **p < 0.01.

Next, the mRNA targets of MCM3AP‐AS1 predicted by starBase and the targets of miR‐193a‐5p predicted by TargetScan were analyzed to determine whether MCM3AP‐AS1 affects the expression of endogenous target genes of miR‐193a‐5p. SENP1 was found to be a potential target of MCM3AP‐AS1/miR‐193a‐5p axis (Figure 5A). Furthermore, the mRNA and the protein expression levels of SENP1 were both down‐regulated in MCM3AP‐AS1‐depleted or miR‐193a‐5p overexpressed CRC cells (Figure 5B–D). We further verified whether SENP1 was the target of miR‐193a‐5p using a double luciferase reporter assay, and the results showed that the luciferase activity of SENP1‐3’UTR was largely inhibited by miR‐193a‐5p; nevertheless, the luciferase activity of SENP1‐3’UTR‐MUT was not affected by miR‐193a‐5p overexpression (Figure 5E). The expression correlations among MCM3AP‐AS1, miR‐193a‐5p, and SENP1 were analyzed using the Broad Institute Cancer Cell Line Encyclopedia (CCLE) dataset. The results showed that miR‐193a‐5p expression was inversely correlated with MCM3AP‐AS1 and SENP1 expression, whereas MCM3AP‐AS1 expression was positively correlated with SENP1 expression in CCLE CRC cell lines (Figure S2). All these data suggest that SENP1 is a target mRNA of MCM3AP‐AS1/miR‐193a‐5p axis.

### 3.5 SENP1 is up‐regulated in CRC tissues

**FIGURE 6:**
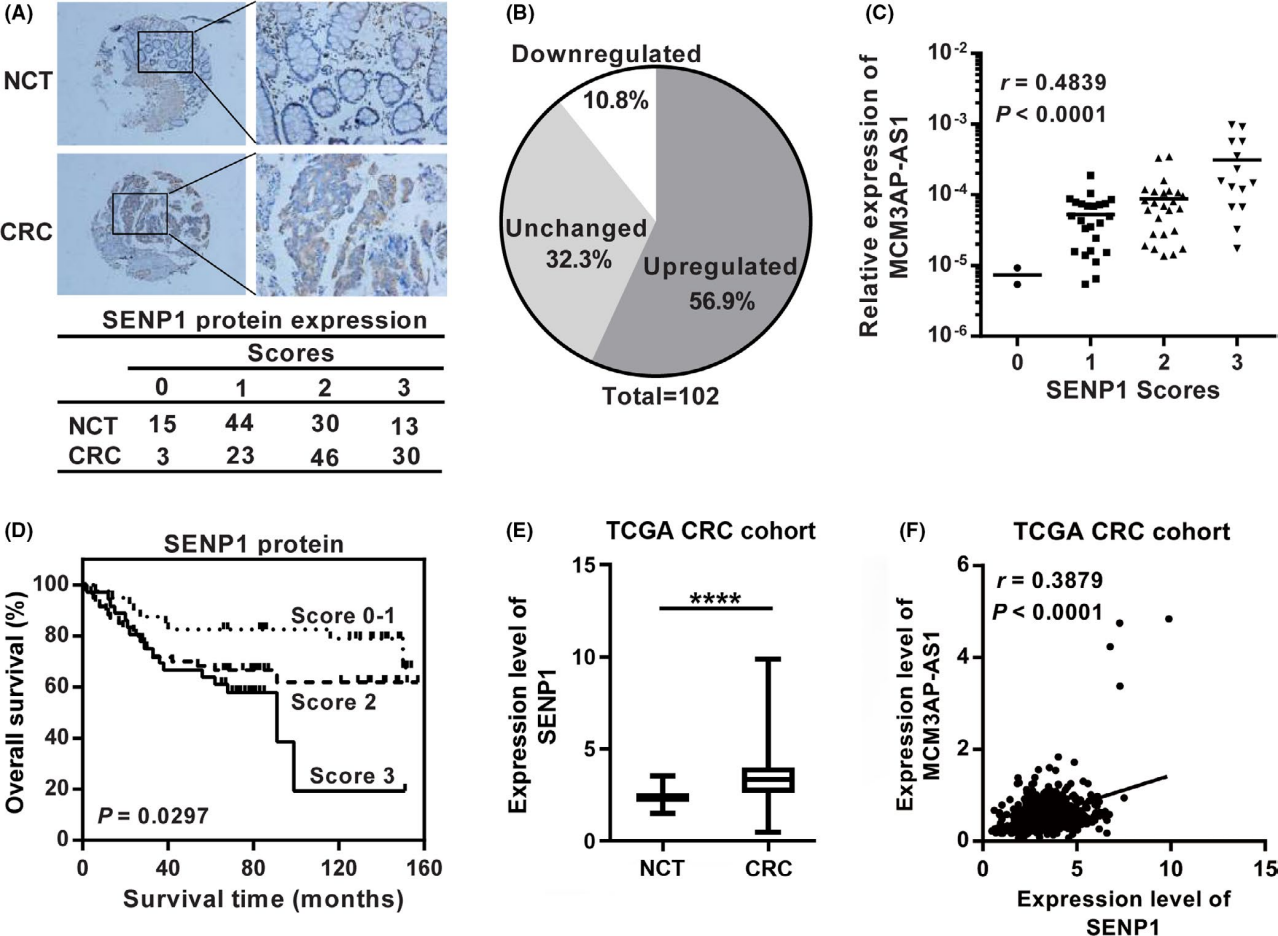
The protein expression of SENP1 was up‐regulated in CRC tissues compared with paired NCTs and positively correlated with the MCM3AP‐AS1 expression. (A) Immunohistochemistry staining of SENP1 in CRC tissues and NCTs. (B) The percentage of upregulated, downregulated and unchanged SENP1 in 102 paired CRC tissues and NCTs. (C) Bivariate correlation analysis between MCM3AP‐AS1 and SENP1 expression in CRC tissues. (D) Kaplan‐Meier survival analysis based on the scores of SENP1 protein expression. (E) The mRNA expression levels of SENP1 of CRC in TCGA database. (F) Bivariate correlation analysis between MCM3AP‐AS1 and SENP1 expression of CRC in TCGA database. ****p < 0.0001.

In order to further demonstrate the relationship between SENP1 and MCM3AP‐AS1, the protein expression of SENP1 in CRC tissues was assessed using IHC (Figure 6A). The results of IHC indicated that compared with paired NCTs, 56.9% (58/102) of tumor tissues showed increased SENP1 expression (Figure 6B). Moreover, the protein expression of SENP1 in human CRC tissues was positively correlated with the expression of MCM3AP‐AS1 (Figure 6C). Kaplan‐Meier survival analysis revealed that the high protein expression level of SENP1 is closely related to the low survival rate of CRC patients (Figure 6D). We also observed an increase in SENP1 mRNA level and a positive correlation with MCM3AP‐AS1 expression in the CRC cohort of TCGA dataset (Figure 6E–F). In summary, these data suggested that SENP1 is a candidate effector of MCM3AP‐AS1.

### 3.6 MCM3AP‐AS1/miR‐193a‐5p/SENP1 axis plays a carcinogenic role in CRC

**FIGURE 7:**
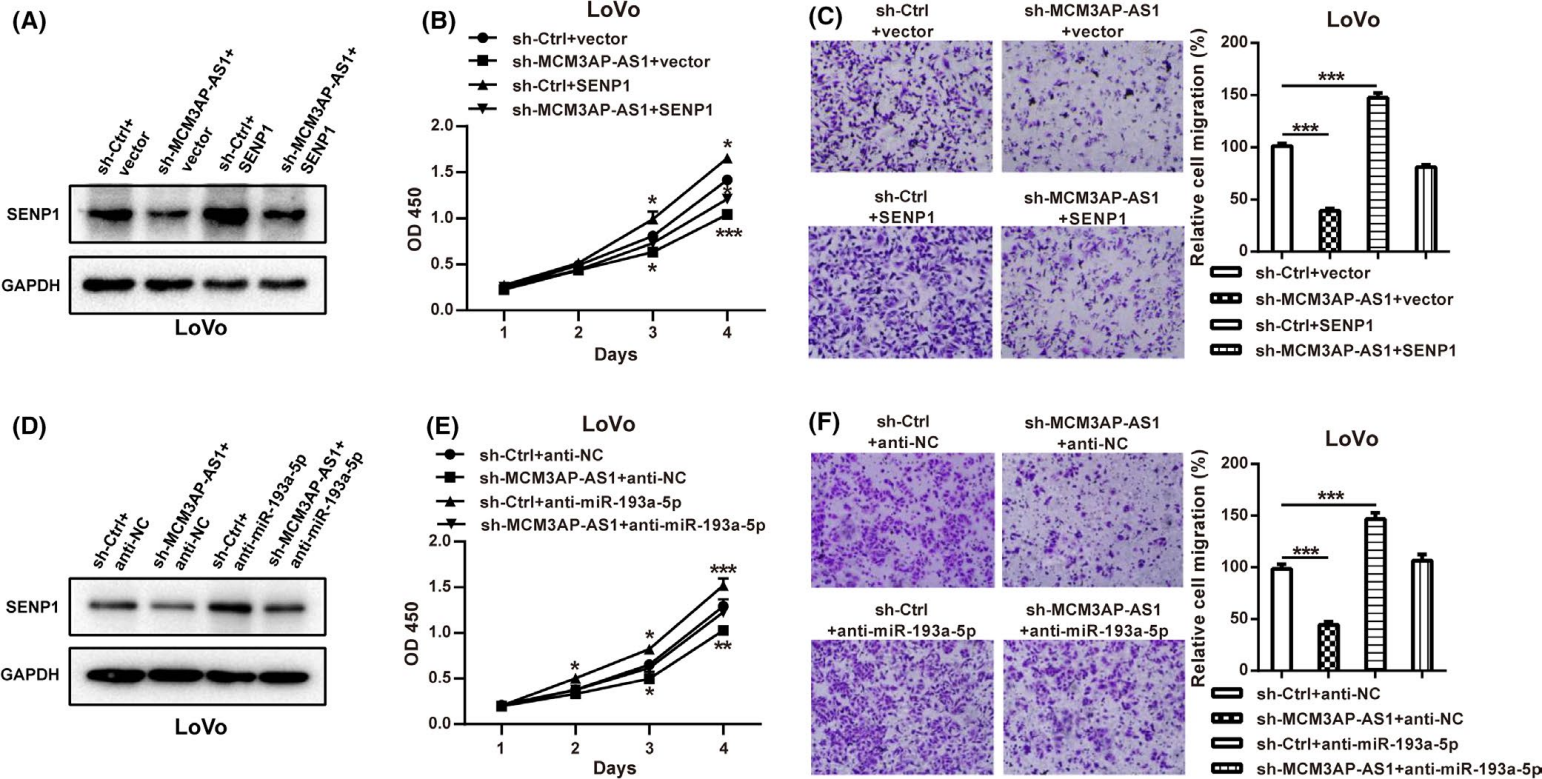
MCM3AP‐AS1 exerted its oncogenic role through the MCM3AP‐AS1/miR‐193a‐5p/SENP1 axis. (A) The protein levels of SENP1 were determined in LoVo cells with stable MCM3AP‐AS1 silencing after transfected with pcDNA3.1‐SENP1 by western blotting. (B, C) The rescuing effects of SENP1 on the decreased proliferation (B) and migration (C) induced by MCM3AP‐AS1 knockdown were detected by CCK8 and transwell assays. (D) The protein levels of SENP1 were determined in LoVo cells with stable MCM3AP‐AS1 silencing after transfected with miR‐193a‐5p inhibitor by western blotting. (E, F) miR‐193a‐5p inhibitor rescued the proliferation (E) and migration‐inhibiting (F) effects of MCM3AP‐AS1 knockdown. *p < 0.05, **p < 0.01, ***p < 0.001.

As described above, we have proved that MCM3AP‐AS1 can sponge miR‐193a‐5p and up‐regulate SENP1 expression. However, whether MCM3AP‐AS1 can promote the development of CRC through miR‐193a‐5p/SENP1 axis needs further verification. The protein levels of SENP1 were restored in LoVo cells with stable MCM3AP‐AS1 silencing after transfected with pcDNA3.1‐SENP1 (Figure 7A). As shown in Figure 7B,C, the reintroduction of SENP1 expression saved the inhibitory effect of MCM3AP‐AS1 knockdown on cell proliferation and migration of CRC cells. Furthermore, the protein levels of SENP1 were also restored in LoVo cells with stable MCM3AP‐AS1 silencing after transfected with miR‐193a‐5p inhibitor (Figure 7D). As shown in Figure 7E,F, suppressing miR‐193a‐5p expression could also reverse the decrease of cell proliferation and metastasis caused by MCM3AP‐AS1 knockdown in CRC cells. Collectively, these data indicated that MCM3AP‐AS1 could play oncogenic functions through the MCM3AP‐AS1/miR‐193a‐5p/SENP1 axis.

## 4 DISCUSSION

CRC is one of the most common malignant tumors of the digestive system and has been associated with poor 5‐year survival rate.^22^ LncRNAs can act as oncogenes or cancer suppressor genes, whose dysregulation is highly associated with the development and progression of CRC. In this work, we identified a new MCM3AP‐AS1/miR‐193a‐5p/SENP1 regulatory axis in CRC.

Recent studies have suggested that MCM3AP‐AS1 could play a key role in human cancers. MCM3AP‐AS1 was originally reported to be overexpressed in glioblastoma^17^ and the up‐regulation of MCM3AP‐AS1 stimulated cell migration and tube formation of clear cell renal cell carcinoma.^23^ MCM3AP‐AS1 could also exert a carcinogenic role in hepatocellular carcinoma and papillary thyroid cancer.^20^, ^24^ We revealed that MCM3AP‐AS1 was aberrantly expressed in CRC tissues and associated with poor prognosis of CRC patients. Up‐regulation of MCM3AP‐AS1 could enhance CRC cell proliferation and metastasis, demonstrating that MCM3AP‐AS1 is an important oncogenic lncRNA in CRC. All these data suggest that MCM3AP‐AS1 is a pan‐cancer oncogene and may be a promising therapeutic target in pan‐cancer.

MCM3AP‐AS1 is located on the opposite strand of MCM3AP on chromosome.^21^ We did not observe an obvious correlation between the expression of MCM3AP and MCM3AP‐AS1 in CRC cell lines (Figure S3A). In addition, MCM3AP level was unchanged in MCM3AP‐AS1‐depleted CRC cells (Figure S3B), suggesting independent expression regulation for the two genes. A key mechanism of cytoplasmic lncRNAs is to act as competing endogenous RNAs (ceRNA). LncRNAs could bind with miRNAs through miRNA response element to regulate the expression of target genes. Previous researches have already confirmed that MCM3AP‐AS1 could bind with several miRNAs, such as miR‐194‐5p, miR‐138‐5p, miR‐211‐5p and miR‐142‐3p.^20^, ^21^, ^24^, ^25^ By a series of assays, we demonstrated that MCM3AP‐AS1 could bind to miR‐193a‐5p and inhibit its function. Although Li et al. have reported that miR‐193a‐5p functions as oncogene in pancreatic cancer,^26^ other studies demonstrated that miR‐193a‐5p could inhibit the progression of CRC, and some other cancers.^27^, ^28^, ^29^ In this study, we also confirmed that miR‐193a‐5p serve as a tumor suppressor in CRC and its function could be inhibited by MCM3AP‐AS1.

We further searched for the targets of MCM3AP‐AS1/miR‐193a‐5p axis and identified that SENP1 was a novel functional target gene of MCM3AP‐AS1/miR‐193a‐5p axis. SENP1 is a deSUMOylation enzyme that plays a critical role in DNA replication, cell cycle, and hypoxic response. The overexpression of SENP1 contributed to chemoradiotherapy resistance in non‐small cell lung cancer.^30^ In addition, SENP1 could also promote renal cell carcinoma tumorigenesis.^31^ In this study, we revealed that SENP1 was overexpressed in CRC tissues and up‐regulated by MCM3AP‐AS1. The increased SENP1 expression was correlated with the overexpression of MCM3AP‐AS1 and associated with shorter survival time in CRC.

A recent study reported that MCM3AP‐AS1 could suppress G1 arrest in CRC through miR‐545/CDK4 axis.^32^ Interestingly, Ma et al reported that MCM3AP‐AS1 is mainly located in the nucleus of cells. However, at the same time, they demonstrated that MCM3AP‐AS1 could promote cell proliferation by sponging miR‐545, which should be observed in cytoplasm.^32^ We observed that MCM3AP‐AS1 was mainly located in the cytoplasm of HCT‐8 and LoVo cells, which was consistent with the observations in other tumors^20^, ^33^ and the result predicted by lncLocator database (Figure S4). It is well known that the localization of a lncRNA determines its possible mechanism and ceRNA is one of the posttranscriptional regulatory mechanisms in the cytoplasm. Therefore, the lncRNAs that are mainly located in the cytoplasm rather than the nucleus are applicable to the mechanism of ceRNA. In addition to confirming the proliferation‐promoting function of MCM3AP‐AS1 in CRC, we also demonstrated the metastasis‐promoting role of MCM3AP‐AS1 in CRC both *in vitro* and *in vivo*.

## 5 CONCLUSION

In conclusion, our study identified that MCM3AP‐AS1 was up‐regulated in CRC and promoted CRC tumorigenesis and progression via MCM3AP‐AS1/miR‐193a‐5p/SENP1 axis. Our results suggest that MCM3AP‐AS1 is a promising new target for the treatment of CRC.

## CONFLICTS OF INTEREST

The authors declare that they have no conflict of interest.

## AUTHORS’ CONTRIBUTIONS

Mingyue Zhou and Zehua Bian performed the experiments and analyzed the data. Bingxin Liu, Yi Zhang, Yulin Cao and Shengbai Sun provided assistance for data acquisition. Kaisa Cui, Jiuming Li and Jia Zhang provided assistance for statistical analysis. Mingyue Zhou, Zehua Bian and Zhaohui Huang prepared the figures and drafted the manuscript. Xue Wang, Chaoqun Li, Surui Yao and Yuan Yin revised the manuscript. Zehua Bian, Bojian Fei and Zhaohui Huang designed the study. All authors approved this manuscript.

## Supporting information

Supplementary Material

## Data Availability

The data that support the findings of this study are available from the corresponding author upon reasonable request.
