# Supplementary material for: Long noncoding RNA MCM3AP‐AS1 enhances cell proliferation and metastasis in colorectal cancer by regulating miR‐193a‐5p/SENP1
Source: Cancer Med. 2021 Mar 8;10(7):2470–81. doi: 10.1002/cam4.3830 (PMC7982620; doi:10.1002/cam4.3830)
Supplement: Supplementary file 1 — Supplementary Material [file CAM4-10-2470-s001.docx]

**Supplementary Tables**

**Supplementary Table S1. Clinicopathologic features of CRC patients**

| **Characteristics** | **Colorectal cancer^a^**  **(n=93)** | **Colorectal cancer^b^**  **(n=102)** |
| --- | --- | --- |
| Ages (years) |  |  |
| < 60 | 52 | 56 |
| ≥ 60 | 41 | 46 |
| Gender |  |  |
| Male | 53 | 59 |
| Female | 40 | 43 |
| Tumor size (cm) |  |  |
| < 5 | 50 | 59 |
| ≥ 5 | 43 | 43 |
| Location |  |  |
| Colon | 48 | 48 |
| Rectum | 45 | 54 |
| Differentiation |  |  |
| Well and moderately | 77 | 82 |
| Poorly | 16 | 20 |
| Invasion depth |  |  |
| T1 | 2 | 3 |
| T2 | 15 | 21 |
| T3 | 44 | 48 |
| T4 | 32 | 30 |
| Lymphatic invasion |  |  |
| Absent | 58 | 64 |
| Present | 35 | 38 |
| Distant metastasis |  |  |
| Absent | 90 | 98 |
| Present | 3 | 4 |
| Tumor stage |  |  |
| I | 19 | 21 |
| II | 37 | 43 |
| III | 33 | 34 |
| IV | 4 | 4 |

^a^: MCM3AP-AS1 analyses were available in 93 of the 131 cases. ^b^: SENP1 expression data were available in 102 of the 131 cases. Both MCM3AP-AS1 analyses and SENP1 expression data were available in 64 of the 131 cases.

**Supplementary Table S2. Primer sequences**

| Primers for real time PCR | | |
| --- | --- | --- |
| Primers | **Sequences-F** | **Sequences-R** |
| MCM3AP-AS1 | TCCCCTCTTGAGCACACTCT | TTCTTGGTTCAGCCCCTTGT |
| SENP1 | CTTGGCTCAGGCGATTTAAGAAC | GAGGTAAAGACTTCGGCTGTTTC |
| β-actin | AGTGTGACGTGGACATCCGCAAAG | ATCCACATCTGCTGGAAGGTGGAC |
| U6 | CTCGCTTCGGCAGCACA | AACGCTTCACGAATTTGCGT |
| miR-193a-5p | ACACTCCAGCTGGGAGTAGAGCGGGCGTT | CTCAACTGGTGTCGTGGAGTCGGCAATTCAGTTGAGACCCAGAA |
| Sequences for gene knockdown | | |
| Si-MCM3AP-AS1-1 | GCUGCUAAUGGCAACACUGATT | UCAGUGUUGCCAUUAGCAGCTT |
| Si-MCM3AP-AS1-2 | CCUUAUGCACAGAGGCAUUTT | AAUGCCUCUGUGCAUAAGGTT |
| Si-MCM3AP-AS1-3 | CCUGUACUGGUCAGGAUUUTT | AAAUCCUGACCAGUACAGGTT |
| Negative control | UUCUCCGAACGUGUCACGUTT | ACGUGACACGUUCGGAGAATT |
| Sh-MCM3AP-AS1 | CCGGGCTGCTAATGGCAACACTGACTCGAGTCAGTGTTGCCATTAGCAGCTTTTTTG | AATTCAAAAAAGCTGCTAATGGCAACACTGACTCGAGTCAGTGTTGCCATTAGCAGC |

**Supplementary Figures**

**

**

**Figure S1.** Relative expression level of MCM3AP-AS1 in CRC tissues and NCTs from GEO dataset. *****P* < 0.0001.

**
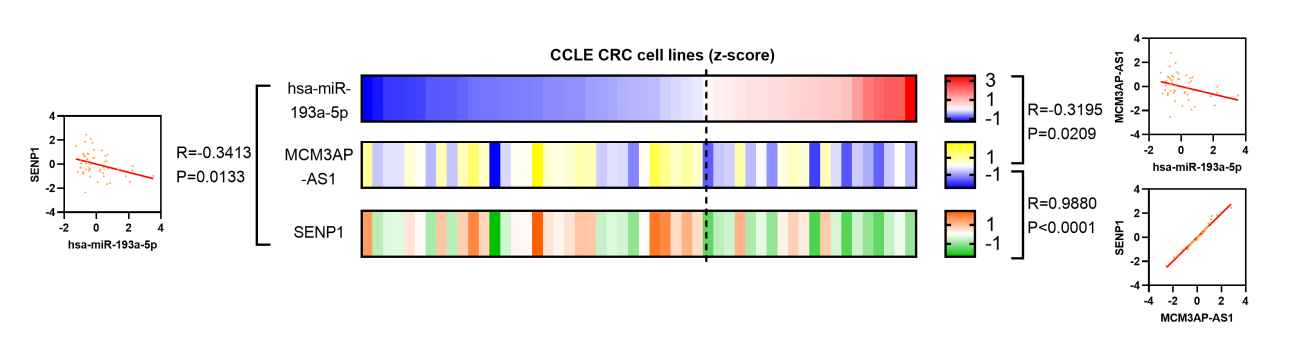
**

**Figure S2.** Expression level of MCM3AP-AS1, miR-193a-5p and SENP1 and their correlation in CCLE CRC cell lines.





**Figure S3.** Expression correlation of MCM3AP and MCM3AP-AS1.(A) Expression level of MCM3AP and MCM3AP-AS1 and their correlation in CCLE CRC cell lines. (B) Relative expression level of MCM3AP in MCM3AP-AS1 silencing LoVo cells.

**
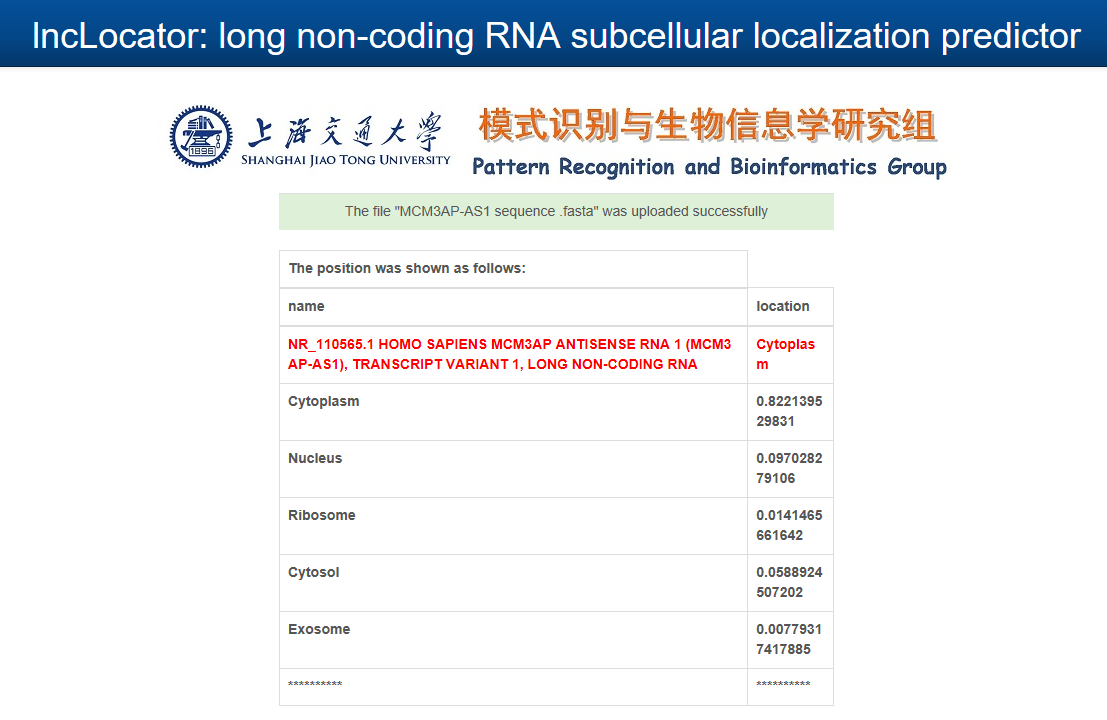
**

**Figure S4.** Subcellular localization of MCM3AP-AS1 predicted by lncLocator database.
